# Incidence and survival of lymphoid neoplasms in Spain, 2002-2013: A population-based study from the Spanish Network of Cancer Registries (REDECAN)

**DOI:** 10.3389/fonc.2022.1046307

**Published:** 2022-11-24

**Authors:** Clàudia Pla, Marta Solans, Alberto Ameijide, Arantza Sanvisens, Marià Carulla, María Dolores Rojas, María Araceli Alemán, Isabel Sáez-Lloret, Cristina Díaz-del-Campo, Ana Isabel Marcos-Navarro, Leire Sainz-de-Aja, Amaia Aizpurua-Atxega, Arantza Lopez-de-Munain, Maria-José Sánchez, Josefina Perucha, Paula Franch, María-Dolores Chirlaque, Marcela Guevara, Jaume Galceran, Susana Merino, Rafael Marcos-Gragera, REDECAN working group, RM-G

**Affiliations:** ^1^ Tarragona Cancer Registry, Cancer Epidemiology and Prevention Service, Sant Joan de Reus University Hospital, Reus, Spain; ^2^ Institut d’Investigació Sanitària Pere Virgili (IISPV), Reus, Tarragona, Spain; ^3^ Research Group on Statistics, Econometrics and Health (GRECS), University of Girona, Girona, Spain; ^4^ Consortium for Biomedical Research in Epidemiology and Public Health (CIBERESP), Madrid, Spain; ^5^ Epidemiology Unit and Girona Cancer Registry, Oncology Coordination Plan, Catalan Institute of Oncology, Girona Biomedical Research Institute Dr. Josep Trueta (IDIBGI), Girona, Spain; ^6^ Josep Carreras Leukaemia Research Institute, Girona, Spain; ^7^ Canary Islands Cancer Registry, Public Health Directorate, Canary Islands Government, Las Palmas, Spain; ^8^ Castellón Cancer Registry, Directorate General of Public Health and Addictions, Valencian Government, Castellón, Spain; ^9^ Ciudad Real Cancer Registry, Health and Social Welfare Authority, Castile−La Mancha, Spain; ^10^ Cuenca Cancer Registry, Health and Social Welfare Authority, Castile−La Mancha, Spain; ^11^ Basque Country Cancer Registry, Basque Government, Vitoria-Gasteiz, Spain; ^12^ Granada Cancer Registry, Andalusian School of Public Health (EASP), Instituto de Investigación Biosanitaria Ibs.GRANADA, University of Granada, Granada, Spain; ^13^ Instituto de Investigación Biosanitaria Ibs.GRANADA, Granada, Spain; ^14^ Department of Preventive Medicine and Public Health, University of Granada, Granada, Spain; ^15^ La Rioja Cancer Registry, Epidemiology and Health Prevention Service, Logroño, Spain; ^16^ Mallorca Cancer Registry, Public Health and Participation Department, Palma de Mallorca, Spain; ^17^ Health Research Institute of the Balearic Islands (IdISBa), Palma de Mallorca, Spain; ^18^ Department of Epidemiology, Regional Health Authority, Instituto Murciano de Investigación Biosanitaria (IMIB)-Arrixaca, Murcia University, Murcia, Spain; ^19^ Navarra Cancer Registry, Navarra Public Health Institute, Pamplona, Spain; ^20^ Epidemiology and Public Health Area, Navarra Institute for Health Research (IdiSNA), Pamplona, Spain; ^21^ Department of Health, Asturias Cancer Registry, Public Health Directorate, Asturias, Spain; ^22^ University of Girona, Girona, Spain

**Keywords:** lymphoid neoplasms, trends, incidence, survival, population-based, Spain

## Abstract

**Introduction:**

The aim of this study was to describe incidence, incidence trends and survival patterns of lymphoid neoplasms (LNs) and its subtypes in Spain in the period 2002-2013 using data from the Spanish Network of Cancer Registries (REDECAN).

**Materials and Methods:**

Data were extracted from 13 Spanish population-based cancer registries. LNs incident cases were codified using the International Classification of Diseases for Oncology, third edition (ICD-O-3) and grouped according to the WHO 2008 classification. Age-standardized incidence rates to the 2013 European standard population (ASIRe) were obtained. Poisson regression models were used to analyze trends in incidence rates and estimate the annual percentage change (APC) for each subtype. The number of cases in Spain for 2023 was estimated by applying the estimated age-specific rates for the year 2023 to the 2023 Spanish population. Observed survival (OS) was estimated by the Kaplan-Meier method and net survival (NS) by the Pohar-Perme method. Sex- and age-specific estimates of 5-year NS were calculated, as well as its changes according to two periods of diagnosis (2002-2007 and 2008-2013).

**Results:**

LNs accounted for 69% (n=39,156) of all hematological malignancies (n=56,751) diagnosed during the period of study. Median age at diagnosis was 67 years (interquartile range (IQR) = 52-77). The overall ASIRe was 34.23 (95% confidence interval (CI): 33.89, 34.57) and showed a marked male predominance in almost all subtypes (global sex ratio = 1.45). During the study period, incidence trends of LNs remained stable (APC: 0.3; 95% CI: -0.1, 0.6), nevertheless some subtypes showed statistically significant variations, such as LNs NOS category (APC: -5.6; 95% CI: -6.8, -4.3). Around 17,926 new cases of LNs will be diagnosed in 2023 in Spain. Survival rates differed considerably across age-groups, while they were similar between men and women. Five- year NS was 62.81% (95% CI: 62.1, 63.52) for all LNs, and varied widely across LNs subtypes, ranging from 39.21% to 90.25%. NS for all LNs improved from the first period of diagnosis to the second one, being 61.57% (95% CI: 60.56, 62.61) in 2002-2007 and 64.17% (95% CI: 63.29, 65.07) in 2008-2013.

**Conclusions:**

This study presents the first complete and extensive population-based analysis of LNs incidence and survival in Spain. These population-based data provide relevant information to better understand the epidemiology of LNs in Southern Europe and it features some useful points for public health authorities and clinicians. However, additional improvements regarding the registration of these hematological neoplasms can be implemented.

## Introduction

Hematological neoplasms are a group of diseases in which the hematopoietic system, the one in charge of the differentiation and proliferation of erythrocytes, thrombocytes, granulocytes and lymphocytes, is involved. The altered proliferation and/or differentiation of the different lineage precursors drives to a wide group of diseases with diverse etiology, presentation and outcomes, and depending on the lineage we find the first stratification of these neoplasms: myeloid neoplasms, lymphoid neoplasms (LNs) and histiocytic/dendritic neoplasms.

LNs, which comprise a heterogeneous group of more than 60 histological subtypes, are ranked as the 6^th^ to 7^th^ most common cancer worldwide (1). Broadly, they are grouped into four main categories: Hodgkin lymphoma, precursor lymphoid neoplasms, mature B-cell neoplasms, and mature T- and NK-cell neoplasms. Changes in our understanding of LNs have resulted in the continuous update of classification schemes over the past 60 years (2). The World Health Organization (WHO) with its first classification of tumors of hematopoietic and lymphoid tissues published in 2001 (3), and subsequently updated in 2008 (4), 2016 (5) and 2022 (6), is currently regarded as the gold standard for the diagnosis and research of LNs. Furthermore, the correspondence established between the WHO classification and the last editions of the International Classification of Diseases for Oncology (ICD-O-3) codes (7), has provided international standards for the registration and epidemiological surveillance of these diseases.

During the last decades, population-based data of LNs has become more available, with multiple studies from hematology-specialized registries (8, 9) or large European and North American datasets (10–14). Specifically in Spain, however, few regional studies have reported incidence and survival data of LNs (15, 16), while there is a lack of subtype-specific data at a nationwide level. Therefore, the aim of this study was to describe incidence and survival patterns of LNs subtypes in Spain over the period 2002-2013, and to estimate the number of LNs expected in 2023, using data from the Spanish Network of Cancer Registries (REDECAN).

## Materials and methods

### Study population

Study data were extracted from 13 Spanish population-based cancer registries (PBCR) (Asturias, Canary Islands –Tenerife and Gran Canaria–, Castellón, Ciudad Real, Cuenca, Euskadi, Girona, Granada, La Rioja, Mallorca, Murcia, Navarra and Tarragona) which together cover 13 provinces and three islands. These PBCR, associated since 2010 in REDECAN (17), provided all LNs, both in pediatric and adult cases, registered from 2002 to 2013 (or the period available between these years) covering almost 26% of the total Spanish population (12,143,157 out of 47,129,783 inhabitants in January 2013) (18).

Quality indicators, which were defined based on the standards of the International Agency for Research on Cancer (IARC) (19), are detailed by PBCR in **Table S1** of the **Supplementary material**. Overall, 96.7% of the cases included were microscopically verified, 5.0% were not otherwise specified (NOS) cases and 1.1% were cases notified to the registry solely based on death certificates (DCO).

### Cases and groupings

Cases were codified using the ICD-O-3 (7) and grouped according to the WHO 2008 classification (4) (**Table 1**). In brief, LNs were grouped into five broad categories: Hodgkin lymphomas, precursor lymphoid neoplasms, mature B-cell neoplasms, mature T-cell and NK-cell neoplasms and lymphoid neoplasms, NOS.

**Table 1:** Crude and age-adjusted incidence rates of lymphoid neoplasms diagnosed in Spain, 2002-2013.

Subtype	ICD-O-3 codes	N. of cases	%	Median Age	CR95% CI	ASIRe95% CI	Sex ratio
**Lymphoid neoplasm, total**		**39,156**	**100.00**	**67**	**31.23 (30.92-31.54)**	**34.23 (33.89-34.57)**	**1.22**
**1) Hodgkin lymphoma**		**3,667**	**9.37**	**36**	**2.92 (2.83-3.02)**	**2.84 (2.74-2.93)**	**1.38**
1.1 Classical Hodgkin lymphoma	9650-9655, 9661-9667	3,455	8.82	36	2.76 (2.66-2.85)	2.67 (2.58-2.76)	1.33
1.1.1 Lymphocyte-rich classical Hodgkin lymphoma	9651	202	0.52	43	0.16 (0.14-0.18)	0.16 (0.14-0.18)	2.37
1.1.2 Nodular sclerosis classical Hodgkin lymphoma	9663-9667	1,906	4.87	31	1.52 (1.45-1.59)	1.43 (1.37-1.5)	1.00
1.1.3 Mixed cellularity classical Hodgkin lymphoma	9652	727	1.86	45	0.58 (0.54-0.62)	0.58 (0.53-0.62)	2.00
1.1.4 Lymphocyte-depleted classical Hodgkin lymphoma	9653-9655	96	0.25	52	0.08 (0.06-0.09)	0.08 (0.06-0.09)	2.43
1.1.5 Classical Hodgkin lymphoma, NOS	9650,9661-9662	524	1.34	45	0.42 (0.38-0.45)	0.42 (0.38-0.46)	1.61
1.2 Nodular lymphocyte predominant Hodgkin lymphoma	9659	212	0.54	38	0.17 (0.15-0.19)	0.17 (0.14-0.19)	2.53
**Non-Hodgkin lymphoma**		**35,489**	**90.64**	**69**	**28.30 (28.01-28.6)**	**31.39 (31.06-31.72)**	**1.21**
**2) Precursor lymphoid neoplasms**		**1,574**	**4.02**	**18**	**1.26 (1.19-1.32)**	**1.31 (1.24-1.37)**	**1.48**
2.1 B-lymphoblastic leukemia/lymphoma	9728, 9811-9819, 9836	508	1.30	17	0.41 (0.37-0.44)	0.42 (0.38-0.46)	1.07
2.2 T-lymphoblastic leukemia/lymphoma	9729, 9837	242	0.62	21	0.19 (0.17-0.22)	0.20 (0.17-0.22)	3.03
2.3 Lymphoblastic leukemia/lymphoma	9727, 9835	824	2.10	18	0.66 (0.61-0.7)	0.69 (0.64-0.74)	1.50
**3) Mature B-cell neoplasms**		**29,429**	**75.16**	**69**	**23.47 (23.2-23.74)**	**26.17 (25.87-26.47)**	**1.19**
3.1 Chronic lymphocytic leukemia/small lymphocytic lymphoma	9670, 9823	6,167	15.75	73	4.92 (4.8-5.04)	5.61 (5.47-5.75)	1.41
3.2 B-cell prolymphocytic leukemia	9833	29	0.07	72	0.02 (0.01-0.03)	0.03 (0.02-0.04)	0.93
3.3 Mantle cell lymphoma	9673	896	2.29	69	0.71 (0.67-0.76)	0.81 (0.76-0.87)	2.28
3.4 Lymphoplasmacytic lymphoma/Waldenström’s Macroglobulinemia	9671, 9761	880	2.25	72	0.70 (0.66-0.75)	0.80 (0.74-0.85)	1.75
3.5 Diffuse large B-cell lymphoma	9675, 9678-9680, 9684, 9688, 9712, 9735, 9737-9738	6,933	17.71	68	5.53 (5.4-5.66)	6.04 (5.9-6.19)	1.16
3.6 Burkitt lymphoma/leukemia	9687, 9826	428	1.09	38	0.34 (0.31-0.37)	0.35 (0.32-0.39)	2.04
3.7 Marginal zone lymphoma	9689, 9699, 9764	2,105	5.38	65	1.68 (1.61-1.75)	1.84 (1.76-1.92)	1.02
3.7.1 Splenic marginal zone lymphoma	9689	403	1.03	68	0.32 (0.29-0.35)	0.36 (0.33-0.4)	0.91
3.7.2 Extranodal marginal zone lymphoma	9699 (excluding C77.0-C77.9)	1,421	3.63	62	1.13 (1.07-1.19)	1.23 (1.16-1.29)	1.06
3.7.3 Nodal marginal zone lymphoma	9699 (C77.0-C77.9)	280	0.72	66	0.22 (0.2-0.25)	0.25 (0.22-0.28)	0.96
3.8 Follicular lymphoma	9597, 9690, 9691, 9695, 9698	4,257	10.87	62	3.39 (3.29-3.5)	3.70 (3.59-3.81)	0.94
3.9 Hairy cell leukemia	9940	274	0.70	61	0.22 (0.19-0.24)	0.24 (0.21-0.26)	3.35
3.10 Plasma cell neoplasms	9731-9734	7,444	19.01	73	5.94 (5.8-6.07)	6.75 (6.59-6.9)	1.06
3.10.1 Solitary plasmocytoma of bone	9731	266	0.68	66	0.21 (0.19-0.24)	0.24 (0.21-0.27)	1.56
3.10.2 Extraosseus plasmocytoma	9734	117	0.30	68	0.09 (0.08-0.11)	0.10 (0.08-0.12)	1.85
3.10.3 Plasma cell myeloma/leukemia	9732-9733	7,061	18.03	73	5.63 (5.5-5.76)	6.41 (6.25-6.56)	1.04
3.11 B-cell lymphoma, unclassifiable, with features intermediate between DLBCL and classical HL	9596	**16**	**0.04**	**46**	**0.01 (0.01-0.02)**	**0.01 (0.01-0.02)**	**1.00**
**4) Mature-T-cell and NK-cell neoplasms**		**2,345**	**5.99**	**62**	**1.87 (1.79-1.95)**	**2.01 (1.93-2.09)**	**1.52**
4.1 Mycosis fungoides/Sezary syndrome	9700, 9701	840	2.15	62	0.67 (0.62-0.72)	0.72 (0.67-0.77)	1.51
4.2 Peripheral T/NK-cell lymphoma	9702, 9705, 9708-9709, 9714-9717, 9724-9726	1,142	2.92	63	0.91 (0.86-0.96)	0.98 (0.92-1.03)	1.63
4.2.1 Peripheral T-cell lymphoma, NOS	9702	492	1.26	66	0.39 (0.36-0.43)	0.42 (0.39-0.46)	1.83
4.2.2 Angioimmunoblastic T-cell lymphoma	9705	177	0.45	69	0.14 (0.12-0.16)	0.16 (0.13-0.18)	1.72
4.2.3 Subcutaneous panniculitis-like T-cell lymphoma	9708	11	0.03	69	0.01 (0-0.01)	0.01 (0-0.01)	1.20
4.2.4 Anaplastic large cell lymphoma, ALK-positive	9714	195	0.50	51	0.16 (0.13-0.18)	0.16 (0.14-0.18)	1.91
4.2.5 Hepatosplenic T-cell lymphoma	9716	11	0.03	46	0.01 (0-0.01)	0.01 (0-0.01)	1.20
4.2.6 Enteropathy-associated T-cell lymphoma	9717	39	0.10	56	0.03 (0.02-0.04)	0.03 (0.02-0.04)	1.79
4.2.7 Primary cutaneous gamma-delta T-cell lymphoma	9726	0	0.00	NA	0.00 (0–0)	0.00 (0–0)	NA
4.2.8 Primary cutaneous T-cell lymphoma, NOS	9709	217	0.55	61	0.17 (0.15-0.2)	0.19 (0.16-0.21)	1.09
4.2.9 Systemic EBV-positive T-cell lymphoproliferative disease of childhood	9724	0	0.00	NA	0.00 (0–0)	0.00 (0–0)	NA
4.2.10 Hydroa vacciniforme-like lymphoma	9725	0	0.00	NA	0.00 (0–0)	0.00 (00)	NA
4.3 Adult T-cell leukemia/lymphoma	9827	15	0.04	76	0.01 (0.01-0.02)	0.01 (0.01-0.02)	0.88
4.4 Extranodal NK/Tcell lymphoma, nasal type	9719	80	0.20	60	0.06 (0.05-0.08)	0.07 (0.05-0.08)	2.08
4.5 T-cell large granular lymphocytic leukemia	9831	61	0.16	73	0.05 (0.04-0.06)	0.05 (0.04-0.07)	1.35
4.6 T-cell prolymphocytic leukemia	9834	33	0.08	64	0.03 (0.02-0.04)	0.03 (0.02-0.04)	0.94
4.7 Aggressive NK cell leukemia	9948	9	0.02	67	0.01 (0-0.01)	0.01 (0-0.01)	0.80
4.8 Primary cutaneous CD30 + T-cell lymphoproliferative disorders	9718	165	0.42	56	0.13 (0.11-0.15)	0.14 (0.12-0.16)	1.09
**5) Lymphoid neoplasms, NOS**	**9590, 9591, 9820, 9832, 9970, 9971, 9760, 9762**	**2,141**	**5.47**	**75**	**1.71 (1.64-1.78)**	**1.90 (1.82-1.98)**	**0.99**

Bold values highlight the main subtypes and its data.

### Statistical analysis

Crude (CR) and age-standardized incidence rates using the 2013 European standard population (ASIRe), were calculated using population data provided by the National Statistics Institute (Instituto Nacional de Estadística—INE) (18) and expressed per 100,000 inhabitant-years. Poisson regression models were used to analyze trends in incidence rates and estimate the annual percentage change (APC) for each subtype of LN. Specific rates by age for the year 2023 were estimated by applying the APCs for the period 2002–2013. The projection of the number of cases of LNs for 2023 was determined by applying to the Spanish population of 2023 the age-specific rates estimated for that same year, assuming a stable incidence trend over time.

For the survival analysis, cases, excluding DCO cases or those diagnosed by autopsy, were followed up until 31 December 2015. Vital status follow-up was carried out using multiple sources of information, such as regional mortality registries, National Death Index, social security database, hospital and primary care records and population censuses, as needed and available in each registry. Observed survival (OS) was estimated by the Kaplan-Meier method and net survival (NS) by the Pohar-Perme estimator (20). The 1-, 3- and 5-year OS and NS and their corresponding 95% confidence interval (CI) are presented for all LNs as a whole and for their subtypes. Survival between groups of age, sex and period of diagnosis was compared using a log-rank type test (21). All analyses were performed using R software (version 3.6.1).

### Ethics approval

This study has been carried out using anonymized data from the participating PBCRs that comprise REDECAN. For their part, the cancer registries comply with the legal regulations in force in Europe and Spain on the Protection of Personal Data. No intervention has been performed in human or animal subjects. Informed consent of the patients is not required for this type of study.

## Results

### Incidence

LNs accounted for 69.0% (n=39,156) of all hematological malignancies (n=56,751) diagnosed in the Spanish population covered by the REDECAN during 2002-2013. Among them, 3,667 (9.37%) were Hodgkin lymphoma, 1,574 (4.02%) precursor lymphoid neoplasms, 29,429 (75.16%) mature B-cell neoplasms, 2,345 (5.99%) mature T-cell and NK-cell neoplasms, and 2,141 (5.47%) lymphoid neoplasms, NOS. **Table 1** lists the number of cases, the percentage over the total of LNs, the median age, the CR and the ASIRe with its 95% CI and sex ratio (male/female) of each subgroup and subtype according to the ICD-O-3 codes. Incidence rates (CR and ASIRe) by sex are shown in **Table S2** of **Supplementary material**.

The ASIRe was 2.84 (95% CI: 2.74, 2.93) for Hodgkin lymphoma, 1.31 (95% CI: 1.24, 1.37) for precursor lymphoid neoplasms, 26.17 (95% CI: 25.87, 26.47) for mature B-cell neoplasms, 2.01 (95% CI: 1.93, 2.09) for mature T-cell and NK-cell neoplasms, and 1.90 (95% CI: 1.82, 1.98) for NOS cases. Median age at diagnosis was 67 years (IQR: 52-77) and there was a marked male predominance: the incidence sex ratio was 1.22 and ranged from 0.91 for splenic marginal zone lymphoma and 0.94 for follicular lymphoma leukemia to 3.03 for T-lymphoblastic leukemia/lymphoma and 3.35 for hairy cell. In both sexes as a whole, the most frequent entities were plasma cell myeloma/leukemia (n=7,061, 18.03%), diffuse large B-cell lymphoma (n=6,933, 17.71%), chronic lymphocytic leukemia/small lymphocytic lymphoma (n=6,167, 15.75%), follicular lymphoma (n=4,257, 10.87%) and classical Hodgkin lymphoma (n=3,455, 8.82%).

Incidence trends of LNs during 2002-2013 are detailed in **Table 2**. The incidence of the LN pool was relatively stable over time (APC: 0.3; 95% CI: -0.1, 0.6), while the main subgroups, with the exception of mature T-cell and NK-cell neoplasms, showed significant variations. Within Hodgkin lymphoma, there was a marked increase of nodular lymphocyte predominant subtype (APC: 6.9%). Incidence of precursor neoplasms decreased slightly, yet within this subgroup, there was a marked increase of B- and T- subtypes to the detriment of NOS cases. Among B-mature cell neoplasms, we evidenced a positive trend of follicular lymphoma (4.0%), marginal zone lymphoma (2.9%), and diffuse large B-cell lymphoma (1.2%), in contrast with the negative trend for chronic lymphocytic leukemia/small lymphocytic lymphoma (-2.2%). Among mature T- and NK-cell neoplasms, there was a positive trend for the subgroup that includes peripheral subtypes other than mycosis fungoides/Sézary syndrome. Finally, the NOS subtype decreased markedly (-5.6%). **Table S3** of **Supplementary material** displays incidence trends stratified by sex, showing no notable differences between sexes.

**Table 2:** Incidence trends of lymphoid neoplasms diagnosed in Spain, 2002-2013.

Subtype	APC (%)	95% CI	
**Lymphoid neoplasm, total**	0.3	(-0.1, 0.6)	
**1) Hodgkin lymphoma**	1.1	(0.1, 2.1)	*
1.1 Classical Hodgkin lymphoma	0,7	(-0.3, 1.8)	
1.2 Hodgkin lymphoma, nodular lymphocyte predominant	6.9	(2.5, 11.5)	*
**Non-Hodgkin lymphoma**	0.2	(-0.2, 0.5)	
**2) Precursor lymphoid neoplasms**	**-2,0**	**(-3.5, -0.5)**	*
2.1 B-lymphoblastic leukemia/lymphoma	4,0	(1.2, 6.8)	*
2.2 T-lymphoblastic leukemia/lymphoma	5.2	(1.2, 9.4)	*
2.3 Lymphoblastic leukemia/lymphoma	-7,5	(-9.4, -5.5)	*
**3) Mature B-cell neoplasms**	0.6	(0.3, 1.0)	*
3.1 Chronic lymphocytic leukemia/small lymphocytic lymphoma	-2,2	(-3.0, -1.5)	*
3.2 B-cell prolymphocytic leukemia	5.7	(-5.6, 18.4)	
3.3 Hairy cell leukemia	-0,8	(-4.4, 2.9)	
3.4 Marginal zone lymphoma	2,9	(1.5, 4.2)	*
3.5 Lymphoplasmacytic lymphoma/Waldenström’s macroglobulinemia	1.0	(-1.0, 3.1)	
3.6 Plasma cell myeloma/leukemia	0.1	(-0.6, 0.8)	
3.7 Follicular lymphoma	4.0	(3.1, 5.0)	*
3.8 Mantle cell lymphoma	-0,7	(-2.7, 1.3)	
3.9 Diffuse large B-cell lymphoma	1.2	(0.5, 2.0)	*
3.10 Burkitt lymphoma/leukemia	-0,8	(-3.7, 2.1)	
3.11 Composite Hodgkin and non-Hodgkin lymphoma	44.3	(17.6, 77.1)	*
**4) Mature-T-cell and NK-cell neoplasms**	**1.3**	**(0.0, 2.6)**	
4.1 Mycosis fungoides/Sézary syndrome	-0,4	(-2.5, 1.7)	
4.2 Other peripheral T- cell and NK-cell lymphoma	2,3	(0.7, 3.9)	*
**5) Lymphoid neoplasms, NOS**	**-5,6**	**(-6.8, -4.3)**	*

Bold values highlight the main subtypes and its data.

* p-value < 0.05.

### Projections for 2023

Predicted incidence rates of LNs subtypes for 2023, overall and by sex, are detailed in **Table S4** of **Supplementary material**. According to our predictions, 17,926 new cases of LNs will be diagnosed in Spain in 2023, of which 1,513 will be Hodgkin lymphoma, 446 precursor lymphoid neoplasms, 14,211 mature B-cell neoplasms, 1,133 mature T-cell and NK-cell neoplasms and 624 lymphoid neoplasms NOS.

### Survival

A total of 38,699 LNs were included in the survival analysis. **Table 3** shows a simplified classification of LNs subtypes with its number of cases, 5-year OS and NS with their 95% CI. A more extensive table that also includes 1- and 3 -year OS and NS is available in **Supplementary material**, **Table S5**. The overall 5-y OS and NS were 56.84% (95% CI: 56.33-57.34) and 62.81% (95% CI: 62.1-63.52), respectively. Within the major categories, Hodgkin lymphoma showed higher 5-y NS (82.6%, 95% CI: 81.25-83.98) compared to non-Hodgkin lymphoma 5-y NS (60.74%, 95% CI: 59.97-61.51), with the rates being higher in mature T-cell and NK-cell neoplasms (64.19%, 95% CI: 61.78-66.7) and mature B-cell neoplasms (62.11%, 95% CI: 61.25-62.99) than in precursor lymphoid neoplasms (54.33%, 95% CI: 51.75-57.03). Regarding specific lymphoma subtypes, the lowest survival rates were observed in plasma cell myeloma/leukemia (39.21%, 95% CI: 37.87-40.61), B-cell prolymphocytic leukemia (47.27%, 95% CI: 29.5-75.74), peripheral T- cell and NK-cell lymphoma (52.12%, 95% CI: 49.18-55.22) and mantle cell lymphoma (52.42%, 95% CI: 48.64-56.51). By contrast, the highest survival rates were estimated for Hodgkin lymphoma nodular lymphocyte predominant (90.25%, 95% CI: 85.79-94.93), hairy cell leukemia (85.89%, 95% CI: 79.87-92.37) and mycosis fungoides/Sézary syndrome (85.76%, 95% CI: 82.02-89.66).

**Table 3:** Five-years observed and net survival of lymphoid neoplasms in Spain.

Subtypes	N	5-year observed survival (%)	95% CI	5-year net survival (%)	95% CI
**Lymphoid neoplasm, total**	**38,699**	**56.84**	**(56.33-57.34)**	**62.81**	**(62.1-63.52)**
**1) Hodgkin lymphoma**	**3,651**	**80.72**	**(79.43-82.04)**	**82.6**	**(81.25-83.98)**
1.1 Classical Hodgkin lymphoma	3,439	80.22	(78.87-81.58)	82.13	(80.72-83.56)
1.2 Hodgkin lymphoma, nodular lymphocyte predominant	212	88.96	(84.68-93.45)	90.25	(85.79-94.93)
**Non-Hodgkin lymphoma**	**35,048**	**54.34**	**(53.8-54.88)**	**60.74**	**(59.97-61.51)**
**2) Precursor lymphoid neoplasms**	**1,464**	**53.8**	**(51.27-56.46)**	**54.33**	**(51.75-57.03)**
2.1 B-lymphoblastic leukemia/lymphoma	474	55.15	(50.78-59.9)	55.59	(51.17-60.4)
2.2 T-lymphoblastic leukemia/lymphoma	235	52.71	(46.59-59.63)	53.13	(46.95-60.13)
2.3 Lymphoblastic leukemia/lymphoma	755	53.33	(49.85-57.06)	53.92	(50.36-57.73)
**3) Mature B-cell neoplasms**	**29,236**	**55.38**	**(54.8-55.97)**	**62.11**	**(61.25-62.99)**
3.1 Chronic lymphocytic leukemia/small lymphocytic lymphoma	6,136	62.67	(61.44-63.93)	74.54	(71.86-77.32)
3.2 B-cell prolymphocytic leukemia	29	43.47	(28.37-66.59)	47.27	(29.5-75.74)
3.3 Hairy cell leukemia	273	77.75	(72.83-83.01)	85.89	(79.87-92.37)
3.4 Marginal zone lymphoma	2,102	76.4	(74.55-78.3)	83.62	(81.16-86.15)
3.5 Lymphoplasmacytic lymphoma/Waldenström’s macroglobulinemia	864	60.88	(57.57-64.38)	69.8	(65.45-74.44)
3.6 Plasma cell myeloma/leukemia	7,324	34.71	(33.59-35.86)	39.21	(37.87-40.61)
3.7 Follicular lymphoma	4,249	77.58	(76.29-78.89)	83.29	(81.78-84.83)
3.8 Mantle cell lymphoma	896	46.81	(43.56-50.3)	52.42	(48.64-56.51)
3.9 Diffuse large B-cell lymphoma	6,930	50.48	(49.29-51.69)	55.58	(54.2-57)
3.10 Burkitt lymphoma/leukemia	417	55.92	(51.33-60.92)	56.91	(52.23-62.01)
3.11 Composite Hodgkin and non-Hodgkin lymphoma	16	81.25	(64.21-100)	83.35	(66.09-105.13)
**4) Mature-T-cell and NK-cell neoplasms**	**2,342**	**58.76**	**(56.77-60.83)**	**64.19**	**(61.78-66.7)**
4.1 Mycosis fungoides/Sézary syndrome	838	78.36	(75.53-81.28)	85.76	(82.02-89.66)
4.2 Other peripheral T- cell and NK-cell lymphoma	1,504	47.84	(45.34-50.48)	52.12	(49.18-55.22)
**5) Lymphoid neoplasms, NOS**	**2,006**	**34.3**	**(32.25-36.47)**	**41.07**	**(38.43-43.9)**

Bold values highlight the main subtypes and its data.

**Figure 1:**
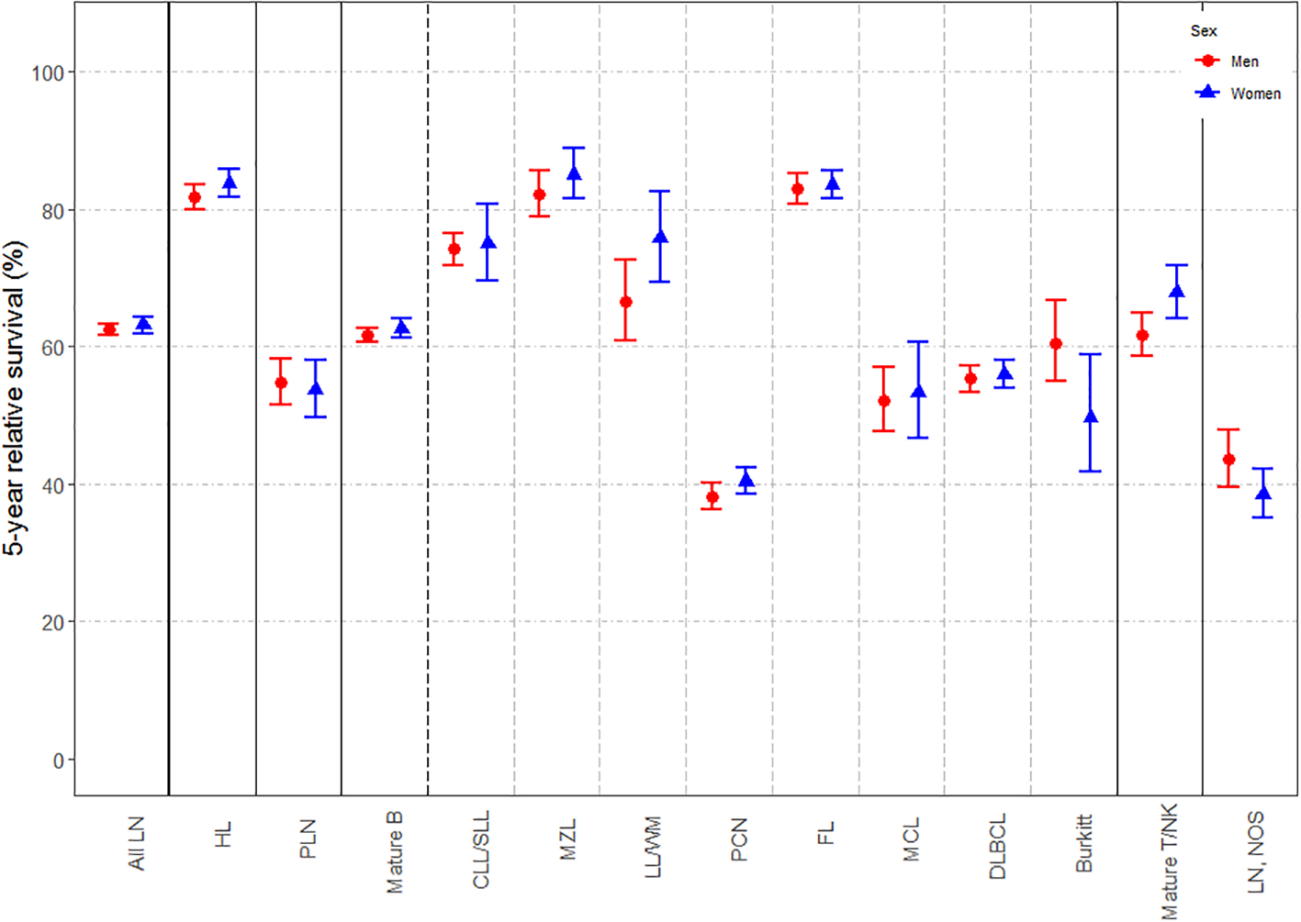
Estimates of 5-year net survival for patients with lymphoid neoplasms diagnosed in 2002-2013, in Spain, according to sex. LN, Lymphoid neoplasm; HL, Hodgkin lymphoma; PLN, precursor lymphoid neoplasms; mature B, mature B-cell neoplasms; CLL/SLL, chronic lymphocytic leukemia/small lymphocytic lymphoma; MZL, marginal zone lymphoma; LL/WM, lymphoplasmacytic lymphoma/Waldenström’s macroglobulinemia; PCN, plasma cell neoplasms; FL, follicular lymphoma; MCL, mantle cell lymphoma; DLBCL, diffuse large B-cell lymphoma; Burkitt, Burkitt lymphoma/leukemia; Mature T/NK, mature T-cell and NK-cell neoplasms; NOS, not otherwise specified.

**Figure 2:**
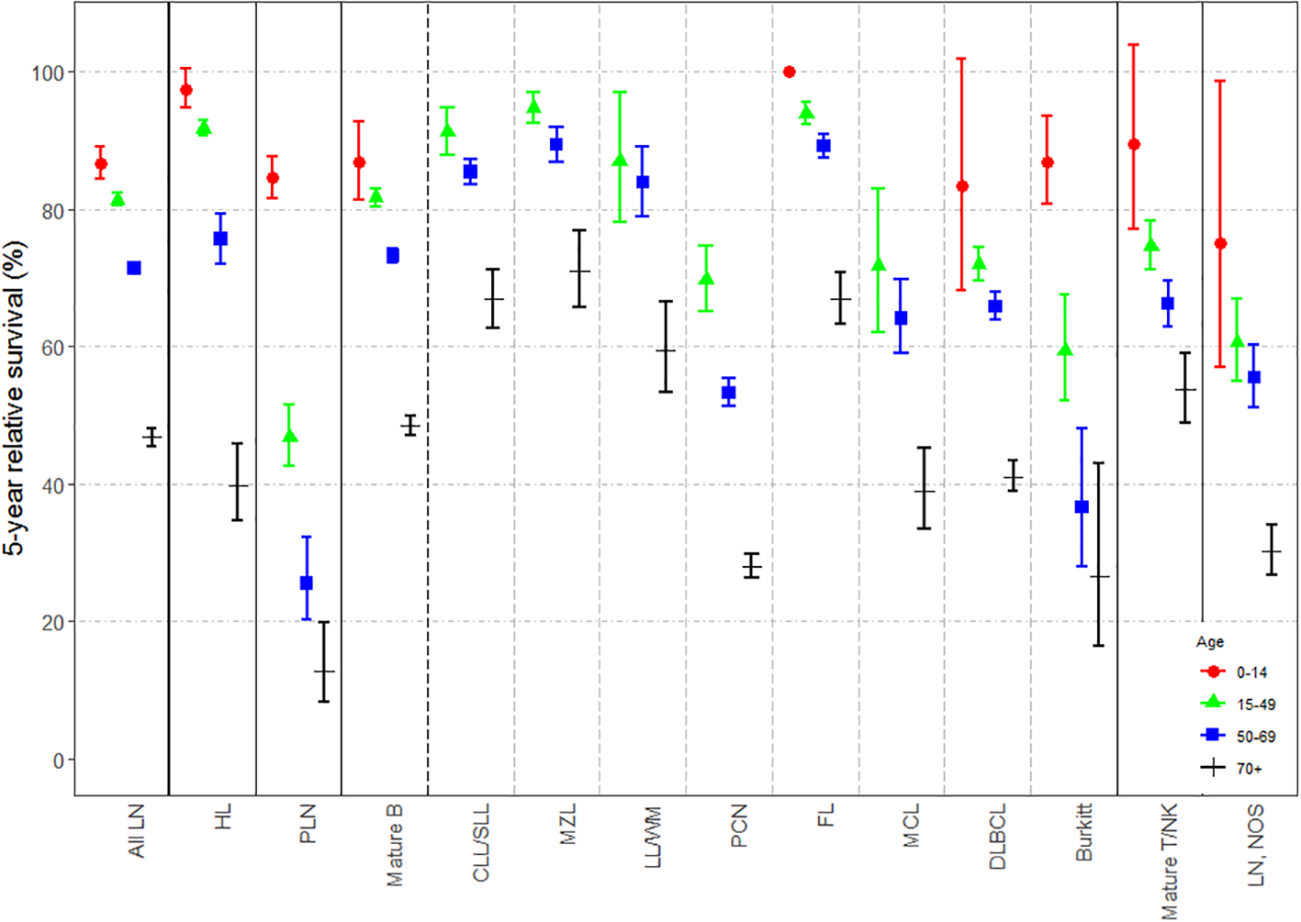
Estimates of 5-year net survival for patients with lymphoid neoplasms diagnosed in 2002-2013, in Spain, according to age-group (0-14, 15-49, 50-69 and 70 or more). LN, Lymphoid neoplasm; HL, Hodgkin lymphoma; PLN, precursor lymphoid neoplasms; mature B, mature B-cell neoplasms; CLL/SLL, chronic lymphocytic leukemia/small lymphocytic lymphoma; MZL, marginal zone lymphoma; LL/WM, lymphoplasmacytic lymphoma/Waldenström’s macroglobulinemia; PCN, plasma cell neoplasms; FL, follicular lymphoma; MCL, mantle cell lymphoma; DLBCL, diffuse large B-cell lymphoma; Burkitt, Burkitt lymphoma/leukemia; Mature T/NK, mature T-cell and NK-celll neoplasms; NOS, not otherwise specified.

**Figure 3:**
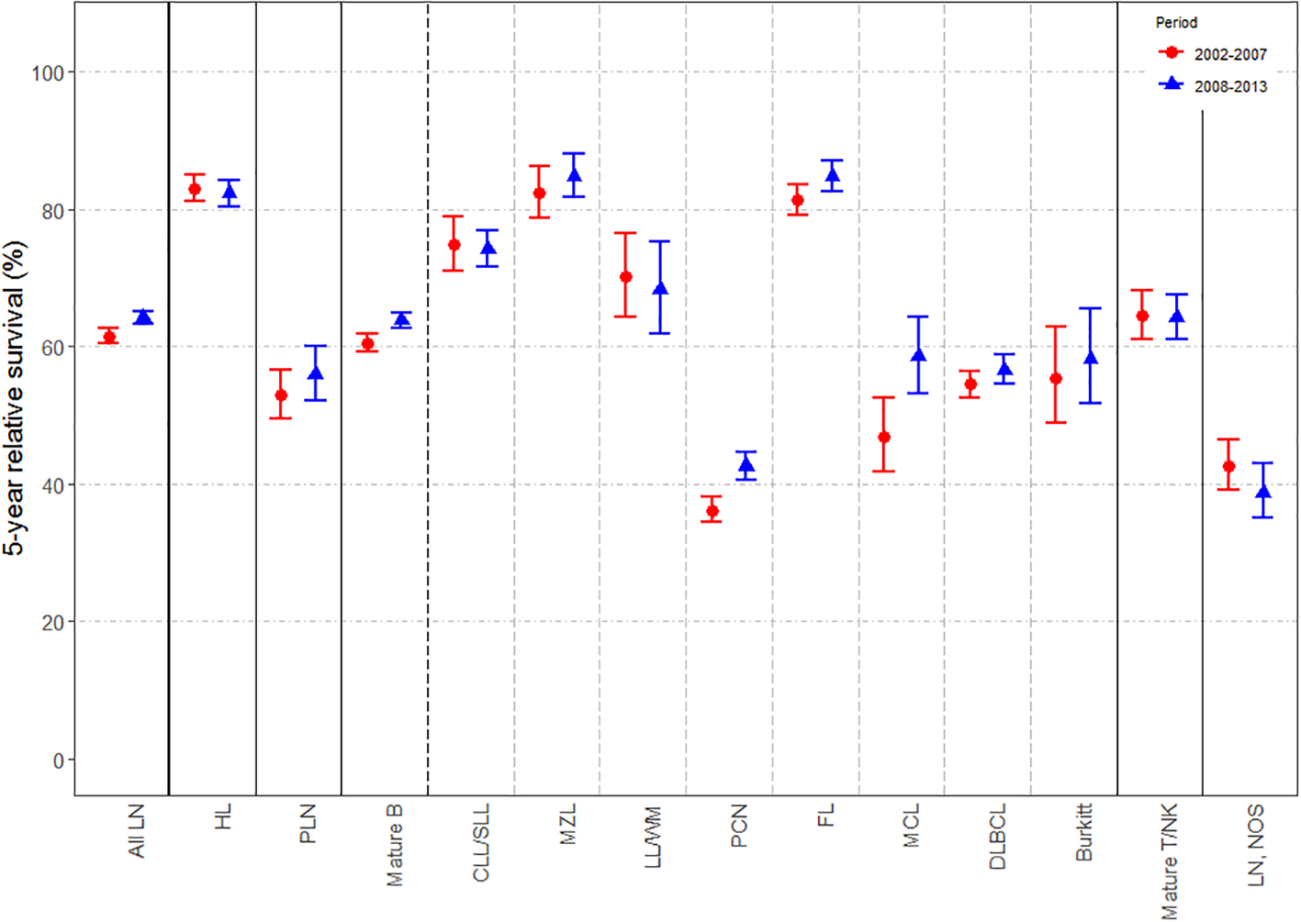
Estimates of 5-year net survival for patients with lymphoid neoplasms diagnosed in 2002-2013, in Spain, according to period of diagnosis (2002-2007 and 2008-2013). LN, Lymphoid neoplasm; HL, Hodgkin lymphoma; PLN, precursor lymphoid neoplasms; mature B, mature B-cell neoplasms; CLL/SLL, chronic lymphocytic leukemia/small lymphocytic lymphoma; MZL, marginal zone lymphoma; LL/WM, lymphoplasmacytic lymphoma/Waldenström’s macroglobulinemia; PCN, plasma cell neoplasms; FL, follicular lymphoma; MCL, mantle cell lymphoma; DLBCL, diffuse large B-cell lymphoma; Burkitt, Burkitt lymphoma/leukemia; Mature T/NK, mature T-cell and NK-celll neoplasms; NOS, not otherwise specified.

Changes in 5-y NS according to sex, age-group and period of diagnosis (i.e. 2002-2007 and 2008-2013) are displayed in **Figures 1–3**, respectively; specific survival values and p-values for log-rank type test are listed in **Tables S6–S8** of **Supplementary material**. Extended versions of these figures for mature T-cell and NK-cell neoplasms subtypes are displayed in **Figures S1–S3** of **Supplementary material**. Slight but statistically significant differences in 5-y NS between sexes were reported globally (**Figure 1**), mostly due to changes in mature B-cell [62.67% (95% CI: 61.21-64.17) for women and 61.65% (95% CI: 60.65-62.66) for men; p-value for log-rank type test <0.001] and mature T/NK-cell neoplasms [67.91% (95% CI: 64.18-71.87) for women and 61.73% (95% CI: 58.63-65) for men; p-value for log-rank type test <0.001]. By contrast, marked differences were found across age-groups (**Figure 2**) and between both periods of diagnosis (**Figure 3**). Five-y NS of all LNs decreased gradually across age-groups, being 86.69% (95% CI: 84.41-89.03) in children (<15 years), 81.4% (95% CI: 80.5-82.3) in 15-49 years, 71.4% (95% CI: 70.54-72.27) in 50-69 years, and 46.83% (95% CI: 45.53-48.17) in those aged 70+ years (p-value for log-rank type test <0.001). This pattern was reported for all LNs subgroups. Regarding the period of diagnosis, we reported an increase of LNs global survival in the second period 2008-2013 (5-y NS: 64.17%; 95% IC: 63.29-65.07) compared to the period 2002-2007 (5-y NS: 61.57%; 95% CI: 60.56-62.61). This was mostly attributed to an improvement of mature B-cell neoplasms survival (p-value for log-rank type test <0.001), more specifically plasma cell neoplasms (p-value for log-rank type test <0.001) and mantle cell lymphoma (p-value for log-rank type test = 0.005).

## Discussion

This paper presents population-based incidence and survival of LNs in Spain during the period 2002-2013. Our study, which includes 39,156 cases diagnosed and registered by the 13 Spanish PBCRs that make up REDECAN, further complements existing European data by providing incidence patterns, projections for 2023 and survival analysis of LNs by subtypes.

Incidence rates for the most frequent subtypes are in line with those reported in large European datasets (10), as well as by hematology-specialized registries from the United Kingdom (Haematological Malignancy Research Network, HMRN) (8). By contrast, we reported lower rates in comparison to France (Côte d’Or) (9), to the United States 2016 projected incidence (14), and to Australia’s data for 1982-2006 (22), presumably due to differences in the study periods and classification. Regarding specific entities, we reported lower rates of marginal zone lymphoma and higher rates of follicular lymphoma when comparing to the HMRN (8), probably due to differences in classification and different completeness of cancer registries. Similarly, we report higher rates of chronic lymphocytic leukemia/small lymphocytic lymphoma and mycosis fungoides/Sézary syndrome, mainly attributable to a higher registration of indolent subtypes by REDECAN rather than a higher actual incidence in Spain. Our data also show higher rates of mantle cell lymphoma which is related to environmental and genetic risk factors like farm life, atopy, and allergy (23).

Incidence of LNs showed a male predominance, with the main exception of follicular lymphoma (sex ratio 0.94), also described in the literature (8, 9). With respect to age at diagnosis, most LNs are diagnosed at advanced ages, with a median age at diagnosis around 67 years. In line with previous studies, cases of precursor lymphoid neoplasms, Hodgkin lymphomas, and Burkitt lymphoma/leukemia were diagnosed at younger ages. In addition, we evidenced that most NOS cases are diagnosed at advanced ages, with an incidence that increases markedly after 70 years. This suggests a decrease in the quality of diagnoses in older people, since they are less likely to receive aggressive diagnostic tests due to comorbidities and/or frailty (24).

The global incidence trend of LN was stable during the study period in accordance with reported results of other Western countries (14, 25), although significant variations were found in specific subtypes. However, the continuous update of the WHO classification of LNs across the period of study hampers the interpretation of these trends. Whilst studies have been shown that computer converted ICD-O-3 historical codes for lymphoma subtypes are generally reliable, and that such agreement is improved when those codes are grouped (26), some changes are hard to overcome. For instance, in 2008 the International Workshop on chronic lymphocytic leukemia changed the definition of the disease, now requiring an absolute B-cell count of 5,000 cells/μL rather than the previous absolute lymphocyte count of 5,000 cells/μL, which caused that many former Rai stage 0 cases now should be considered as a pre-malignant condition (monoclonal B-cell lymphocytosis) (27). This may explain the negative trend in the incidence of chronic lymphocytic leukemia/small lymphocytic lymphoma evidenced in our study. On the other hand, we reported a marked decrease in the incidence of NOS cases across the period of study. This might be attributed to more specific clinical diagnoses and also to improved coding in Spanish PBCRs. These improvements in coding are probably due to the training courses organized by REDECAN and given to registry professionals, in particular hematological neoplasm coding courses held during the study period.

As expected, 5y-NS was widely different across LNs subtypes, and our results are broadly consistent with those reported by specialized registries in the UK (8), France (9), and in European (HAEMACARE, EUROCARE-5) (11–13) and North American datasets (28). However, the survival estimates that we present are higher than those reported by studies using the HAEMACARE database (11). These differences can be due to the HAEMACARE’s study period (2000–2002), prior to the appearance of new treatment lines and more accurate diagnostic techniques that significantly improved survival, and to the use of the Hakulinen method for estimating survival instead of the Pohar-Perme method (29). By contrast, our results for most subtypes were lower than those presented by the HMRN (8).

Overall, survival rates differed considerably across age-groups, a well-established prognostic factor for most cancers (30). The poor survival of elderly patients with LN is mainly attributed to the presence of comorbidities or to their frail status, which hampers firstly, seeking for a specific diagnosis, and subsequently, the application of several treatment protocols, such as several chemotherapy regimens or stem-cell transplantation (31–33). On the other hand, we evidenced differences in the survival of mature B, T and NK-cell neoplasms between sexes that would be related to the impact of chromosomal and hormonal control of immunity (34), among other factors, and its consequent cancer evolution and response to treatment (35, 36). Finally, NS for all LNs improved significantly during the period of study, which was mostly attributed to a better prognosis of mature B-cell neoplasms, particularly in plasma cell neoplasms and mantle cell lymphoma. This could be related to changes in treatment patterns, such the introduction of rituximab during 2004-2005 for mantle cell lymphoma, and the application of novel agents (thalidomide, bortezomib, lenalidomide) together with autologous stem cell transplantation in 2009 for multiple myeloma (37, 38). The favorable effect on survival of these therapeutic changes were also reported by European PBCRs (39–41).

According to our projections, we expect 17,926 new cases of LNs in 2023. However, these data take into account neither the changes in the WHO classification (new updates were launched in 2016 and 2022) (5, 6), nor the impact of coronavirus disease 19 (COVID-19). Regarding the latter, according to multiple studies, the COVID-19 pandemic may have caused significant delays in diagnosis and initiation of treatment in many cancer sites. This may lead to a subsequent increase in the number of cases diagnosed in more advanced stages, with implications on survival, quality of life, and economic costs (42–44). Finally, the projections presented here could be underestimated due to the continuous improvement in the capture of hematological neoplasms by PBCRs and the development in recent years of more effective diagnostic tools. However, the methods and strategy used in data analysis provide a reasonably accurate estimate of the current incidence of LNs in Spain, based on the greatest population coverage currently provided by cancer registries in this country.

Some limitations must be considered when interpreting our data. First, changes in the classifications and diagnostic criteria of the subtypes studied hamper the interpretation of our results, as well as comparisons with previous studies. In addition, lack of homogeneity among REDECAN registries, since there is no centralized pathology and clinical review, can lead to problems of quality and completeness. For instance, this may have implications in the percentage of NOS cases in all provinces or affect the estimates of subtypes such as chronic lymphocytic leukemia or Waldenström’s macroglobulinemia, which have been shown to be underreported by PBCR. Nonetheless, during the last years, REDECAN has implemented training programs to improve the coding and registering of hematological neoplasms in non-specialized Spanish registries, following the example of the French Network of Cancer Registries (FRANCIM) (45). In addition, some LNs subtypes, such as mature T-cell and NK-cell neoplasms, are exceptionally rare; therefore, our estimates may be less robust for those entities. Finally, lack on information on disease stage at diagnosis and therapeutic patterns makes interpretation of survival rates difficult. High-resolution studies collecting such data would provide further evidence on the effectiveness of new treatments, as well as to explain geographic variations across Europe (12).

## Conclusions

This study presents the most comprehensive population-based analysis of LNs incidence and survival in Spain. It features some useful points for public health authorities and clinicians, such as the evolution of the incidence of several subtypes, and the expected cases in 2023 based on these data. Survival for all cases as a whole improved along the period of study, although rates are still poor for several subtypes. This study also provides information on the quality of the registries, suggesting, with a negative trend of NOS cases, improvements in the registration and/or a more specific diagnosis, although there is still room for improvement.

## Data availability statement

The raw data supporting the conclusions of this article will be made available by the authors, without undue reservation.

## REDECAN Working Group

REDECAN Working Group: Girona Cancer Registry – Coordination of hematology neoplasms in REDECAN: RM-G, Montse Puigdemont, Anna Vidal Vila, AS, Andrés Castillo-Bonilla; Tarragona Cancer Registry – Data coordination in REDECAN: MC, AA, CP, and JG; Álava Cancer Registry: AL-d-M, Patricia Sancho, María Luisa Iruretagoyena**;** Albacete Cancer Registry: Cristina Ramírez; Asturias Cancer Registry: SM, Virginia Menéndez García, Marta Rodríguez Camblor; Bizkaia Cancer Registry: Visitación de Castro, Marta De La Cruz, Joseba Bidaurrazaga; Castellón Cancer Registry: Consol Sabater Gregori, Isabel Sáez Lloret, Ana Vizcaíno Batllés, Xavier Peñalver Herrero; Ciudad Real Cancer Registry: CD-d-C; Cuenca Cancer Registry: AM, Rosario Jimenez Chillarón; Gipuzkoa Cancer Registry: AA; Gran Canaria Cancer Registry: MDR, Emilio De Miguel, María Carmen Gabas*;* Granada Cancer Registry: MJS, Daysi Yoe-Ling Chang-Chan; Miguel Rodríguez Barranco; La Rioja Cancer Registry: María Isabel Palacios, Enrique Ramalle; Mallorca Cancer Registry: Paula Franch, Patricia Ruiz Armengol, Carmen Sánchez Contador; Murcia Cancer Registry: MDC, Antonia Sánchez Gil, Ricardo José Vaamonde; Navarra Cancer Registry: MG, Eva Ardanaz; Tenerife Cancer Registry: MAA, Leonor Olga Velázquez, María Magdalena Ramos Marrero; Castilla y León Cancer Registry: Pilar Gutiérrez, Rufino Álamo, Lorena Estévez; Registro Español de Tumores Infantiles: Adela Cañete, Elena Pardo, Rafael Peris Bonet; Registro de Tumores Infantiles y Adolescentes de la Comunitat Valenciana: Consol Sabater Gregori, Ana Vizcaino Batllés, Fernando Almela Vich.

## Author contributions

RM-G, MS, and CP contributed to the study conception and design. Data collection was performed by CP, AS, MC, MDR, MAA, IS-L, CD-d-C, AIM-N, LS-d-A, AA-A, AL-d-M, MJS, JP, PF, MDC, MG, JG, SM, and RM-G. Data analysis was performed by AA and AS. The first draft of the manuscript was written by CP. All authors contributed to the article and approved the submitted version.

## Funding

This work was partially funded by the Josep Carreras Leukaemia Research Institute (Grant No.: FIJC1100).

## Conflict of interest

The authors declare that the research was conducted in the absence of any commercial or financial relationships that could be construed as a potential conflict of interest.

## Publisher’s note

All claims expressed in this article are solely those of the authors and do not necessarily represent those of their affiliated organizations, or those of the publisher, the editors and the reviewers. Any product that may be evaluated in this article, or claim that may be made by its manufacturer, is not guaranteed or endorsed by the publisher.
